# Colorectal cancer stages transcriptome analysis

**DOI:** 10.1371/journal.pone.0188697

**Published:** 2017-11-28

**Authors:** Tianyao Huo, Ronald Canepa, Andrei Sura, François Modave, Yan Gong

**Affiliations:** 1 Department of Health Outcomes & Policy, College of Medicine, University of Florida, Gainesville, Florida, United States of America; 2 Information Technology and Services, University of Florida, Gainesville, Florida, United States of America; 3 Department of Pharmacotherapy and Translational Research and Center for Pharmacogenomics, College of Pharmacy, University of Florida, Gainesville, Florida, United States of America; 4 University of Florida Health Cancer Center, Gainesville, Florida, United States of America; Sapporo Ika Daigaku, JAPAN

## Abstract

Colorectal cancer (CRC) is the third most common cancer and the second leading cause of cancer-related deaths in the United States. The purpose of this study was to evaluate the gene expression differences in different stages of CRC. Gene expression data on 433 CRC patient samples were obtained from The Cancer Genome Atlas (TCGA). Gene expression differences were evaluated across CRC stages using linear regression. Genes with p≤0.001 in expression differences were evaluated further in principal component analysis and genes with p≤0.0001 were evaluated further in gene set enrichment analysis. A total of 377 patients with gene expression data in 20,532 genes were included in the final analysis. The numbers of patients in stage I through IV were 59, 147, 116 and 55, respectively. *NEK4* gene, which encodes for NIMA related kinase 4, was differentially expressed across the four stages of CRC. The stage I patients had the highest expression of *NEK4* genes, while the stage IV patients had the lowest expressions (p = 9*10^−6^). Ten other genes (*RNF34*, *HIST3H2BB*, *NUDT6*, *LRCh4*, *GLB1L*, *HIST2H4A*, *TMEM79*, *AMIGO2*, *C20orf135 and SPSB3*) had p value of 0.0001 in the differential expression analysis. Principal component analysis indicated that the patients from the 4 clinical stages do not appear to have distinct gene expression pattern. Network-based and pathway-based gene set enrichment analyses showed that these 11 genes map to multiple pathways such as *meiotic synapsis* and *packaging of telomere ends*, etc. Ten of these 11 genes were linked to Gene Ontology terms such as *nucleosome*, *DNA packaging complex* and *protein-DNA interactions*. The protein complex-based gene set analysis showed that four genes were involved in H2AX complex II. This study identified a small number of genes that might be associated with clinical stages of CRC. Our analysis was not able to find a molecular basis for the current clinical staging for CRC based on the gene expression patterns.

## Introduction

Colorectal cancer (CRC) is the third most common cancer and the second leading cause of cancer-related deaths in the United States [1]. Among the five subtypes of CRC (adenocarcinomas, carcinoid tumors, gastrointestinal stromal tumors, lymphomas and sarcomas), adenocarcinomas are the most common (95% of all CRCs). Currently the staging of CRC, referred to as clinical staging, is based on results of physical exams, biopsies, and imaging tests (CT or MRI scan, X-rays, PET scan, etc.). The criteria of staging are based on: 1) how far the cancer has grown into the wall of the intestine; 2) whether it has reached nearby structures; and 3) whether it has spread to the nearby lymph nodes or to distant organs. The results of surgery can be combined with clinical staging to determine the pathologic stages. The most often used CRC staging system is the AJCC cancer staging manual developed by American Joint Committee on Cancer (AJCC), based on conditions of primary tumor (T), regional lymph nodes (N) and distant metastasis (M) [2]. The earliest stage cancers are called stage 0, then range from stage I through IV, with additional sub-stages identified with the letters A, B and C [3].

Several genes, such as *WNT*, *WAPK/PI3K*, *TGF-β*, *TP*, have been associated with CRC. For instance, mutations in adenomatous polyposis col (APC) gene, a tumor suppressor gene, were found to be responsible for familial adenomatous polyposis and then further developed to CRC [4]. MisMatch Repair system genes such as *MLH1* and *MSH2* gene were found to be associated with Lynch syndrome, the most frequent form of hereditary CRC [5, 6]. Further, a 12-gene recurrence score assay has been developed as a prognostic factor in stage II-III colon or rectal carcinoma [7–9]. Even though many genes have been associated with an increased risk of CRC, the genetic differences across different stages of CRC have not been clearly identified. So far, only one study had assessed the gene expression levels of three candidate genes (*MMP9*, *MMP28* and *TIMP1*) across CRC stages and found no statistically significant differences based on the stage of CRC [10]. There have been no studies in the literature comparing the gene expression levels in the entire transcriptome across CRC stages. The purpose of this study is to explore transcriptome-wide gene expression differences across different stages of CRC followed by gene ontology, gene set network analysis approaches based on the publicly available RNAseq dataset in The Cancer Genome Atlas (TCGA) [11].

## Materials and methods

### Data acquisition

The Cancer Genome Atlas (TCGA) (http://cancergenome.nih.gov/) is a joint effort between the National Cancer Institute (NCI) and the National Human Genome Research Institute (NHGRI) to facilitate the sharing of data and speed up cancer research [11, 12]. The Eli and Edythe L. Broad (Broad) Institute of MIT and Harvard is a joint venture between both institutions and several area hospitals (https://www.broadinstitute.org/about-us). Their “FireHose” project ingests, aggregates, standardizes, and processes TCGA data via automated pipelines in an attempt to accelerate analysis and discoveries (https://confluence.broadinstitute.org/display/GDAC/Rationale).

The Broad Institute has established pipelines for processing each TCGA dataset and the outputs from each stage of the pipeline are made available as a versioned set. Illumina HiSeq expression data was processed by Broad Institute to output both reads per kilobase per million mapped reads (RPKM) expression values [13] and RNA-seq by Expectation-Maximization (RSEM) values [14] normalized to “upper quartile count at 1000”. TCGA clinical data and expression data were manually downloaded from the Broad Institute (TCGA data version 2016_01_28) via the firebrowse.org website.

(http://firebrowse.org/?cohort=COADREAD&download_dialog=true). The code used to download the data can be accessed here: https://github.com/indera/crc_transcriptome_analysis.

### Data merging

Using Python 2.7.10 and version 0.19.0 of the Pandas module, the expression data from the Broad Institute was read into a Pandas dataframe, transposed, and re-saved. The clinical data were also transposed in the same manner. Additionally, in order to cut down on the size of the data and number of components of interest, only a subset of the columns from the clinical data were kept for the analysis. These included common demographic data such as patient gender, race, ethnicity, and age; clinical data such as cancer stage, associated International Classification of Diseases (ICD) 10 codes, presence of polyps, whether analysis had been done for common mutations such as KRAS and BRAF; and finally, approximately 85 different aliquot identifiers from the TCGA dataset itself.

Matching of clinical data with expression data was performed using TCGA's "hybridization REF" identifier from the expression data and searching against the aliquot identifiers present in the clinical data. Eventually, 377 patients with gene expression data from 20,532 genes were included in the final analysis.

### Differential expression analysis

Gene expression differences were evaluated across the disease stages using linear regression. The standard deviation of the gene expression level for each gene was computed. The genes with standard deviation of zero, which indicates no change in the gene expression, were removed from further analysis. To select top genes that are differentially expressed across cancer stages, a linear regression model was performed for each gene to test the trend in gene expression with increasing cancer stages. The analyses adjusted for age, gender and race/ethnicity of the patients. Genes with p ≤0.0001 were considered suggestive and the expression level by cancer stages were presented for these genes. Analyses were performed using R version 3.3.1 and SAS 9.4 (Cary, NC).

### Principal component analysis

In order to identify gene expression pattern of the selected CRC samples across different stages, all the genes with p≤0.001 in the linear model analysis were included in the principal component analysis using SAS. Ten principal components (PCs) were identified and the first two PCs were plotted according to the staging status of the CRC patients.

### Gene annotation and gene set enrichment analysis

Genes with expression difference of p ≤ 0.0001 were evaluated further in gene annotation using DAVID [15]. Then the gene IDs and official gene names were used for further analysis. ConsensusPathDB tool [16, 17] was then used to perform network-based and pathway-based analyses on these top genes. ConsensusPathDB consists of a comprehensive collection of human, mouse and yeast molecular interaction data integrated from 32 different public repositories and a web interface with a set of computational methods and visualization tools to explore these data (http://consensuspathdb.org). This tool applies computational methods for statistical over-representation and enrichment analysis and reports network modules, pathways and functional information that are significantly enriched by any given gene list. ConsensusPathDB provides 4 types of predefined annotation gene sets: neighborhood-based entity sets (NESTs) which includes protein-protein interactions, biochemical interactions, gene regulatory and genetic interactions, protein complexes, pathways (including metabolic, signaling and gene regulatory pathways) and GO terms [16]. For computing the significance of the enrichment of the annotation sets with respect to user-input gene list, this tool applies Wilcoxon’s matched-pairs signed-rank test.

## Results

### Demographics

The TCGA database contains clinical information for 629 patients but only 396 unique patients have both gene expression data and clinical data. The numbers of patients with CRC in stage I through IV were 59, 147, 116 and 55 respectively and 19 patients did not have stage information and there were no patients in the stage 0. The mean age of these patients was 64 ± 12 years. Further, 46.4% were women, 69.2% were white, 16.2% were Black/African American, 14.6% were Asian, American Indian/Alaska Native and of unspecified race, and 1.1% were Hispanics. From a clinical standpoint, 76.7% had colon cancer and 23.3% had rectal cancer. The demographic and relevant clinical information of these patients stratified by CRC stage are summarized in **Table 1.** The final analysis included 377 patients with clinical data including staging information and gene expression in 20,532 genes.

**Table 1:** Demographics of patients by CRC cancer stages.

Characteristic	Stage I	Stage II	Stage III	Stage IV	Total
	59 (14.89%)	147 (37.12%)	116 (29.29%)	55 (13.88%)	377 (100%)
Age Mean, SD	65 ± 12	67 ± 12	63 ± 13	60 ± 13	64 ± 13
Height Mean, SD (cm)	172 ± 10.8	166.9 ± 12.8	169.0 ± 10.8	171.8 ± 10.9	169.1 ± 11.8
Weight (Kg)	83.1 ± 19.7	77.8 ± 23.3	81.4 ± 20.1	80.6 ± 17.7	80.3 ± 21.2
BMI	28.1	28.0	28.5	27.3	28.1
Sex					
Female	25	72	54	24	175 (46.4%)
Male	34	75	62	31	202 (53.6%)
Vital Status					
Alive	57	133	103	39	332 (88.1%)
Dead	2	14	13	16	45 (11.9%)
Race					
White	43	93	86	39	261 (69.2%)
Black/African American	8	20	22	11	61 (16.2%)
Other	8	34	8	5	55 (14.6%)
Ethnicity					
Hispanic or Latino	0	1	1	2	4 (1.1%)
Not Hispanic or Latino	49	121	105	47	322 (85.4%)
Other	10	25	10	6	51 (13.5%)
Cancer Type					
Colon	46	119	83	41	289 (76.7%)
Rectal	13	28	33	14	88 (23.3%)

SD: standard deviation. Continuous variables were summarized as mean and SD and categorical variables were summarized as number (%).

### Linear model for gene expression

**Fig 1:**
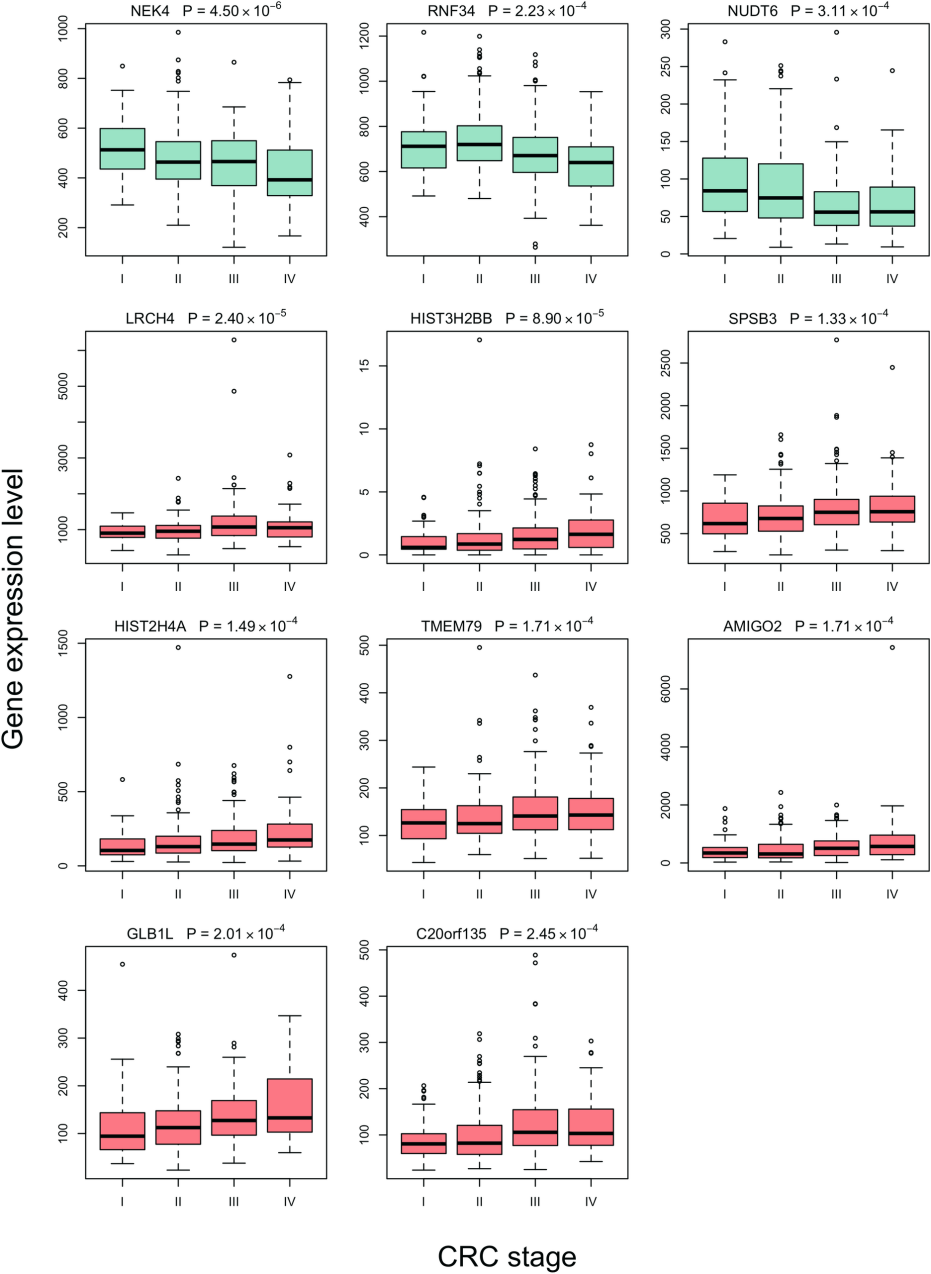
Top gene expression levels by CRC cancer stage.

Eleven genes had p ≤0.0001 in the differential gene expression analysis according to the clinical staging. *NEK4* gene, which encodes for NIMA related kinase 4, was differentially expressed across the four stages of CRC. The samples from the stage I patients had the highest expression of *NEK4* genes, while the stage IV had the lowest expressions (p = 4.50*10^−6^) (**Table 2**, **Fig 1**). Ten other genes had p value of 0.0001 in the unadjusted differential expression analysis including two with decreasing gene expression levels in more advanced CRC stages (*RNF34 and NUDT6) and eight with increasing gene expression levels in more advanced CRC stages (LRCH4*, *HIST3H2BB*, *SPSB3*, *HIST2H4A*, *TMEM79*, *AMIGO2*, *GLB1L and C20orf135*) (**Table 2, Fig 1**).

**Table 2:** The top genes in the linear regression analysis.

Gene	Gene ID	Gene Full Name	P (unadjusted)	P (adjusted)
*NEK4*	6787	NIMA related kinase 4	9.00E-06	4.50E-06
*LRCH4*	4034	leucine rich repeats and calponin homology domain containing 4	1.00E-04	2.40E-05
*HIST3H2BB*	128312	histone cluster 3 H2B family member b	1.00E-04	8.90E-05
*SPSB3*	90864	splA/ryanodine receptor domain and SOCS box containing 3	1.00E-04	1.33E-04
*HIST2H4A*	8370	histone cluster 2 H4 family member a	1.00E-04	1.50E-04
*TMEM79*	84283	transmembrane protein 79	1.00E-04	1.71E0-4
*AMIGO2*	347902	adhesion molecule with Ig like domain 2	1.00E-04	1.71E0-4
*GLB1L*	79411	galactosidase beta 1 like	1.00E-04	2.00E-04
*RNF34*	80196	ring finger protein 34	1.00E-04	2.23E-04
*C20orf135*	140701		1.00E-04	2.40E-04
*NUDT6*	11162	nudix hydrolase 6	1.00E-04	3.10E-04

### Principal component analysis

**Fig 2:**
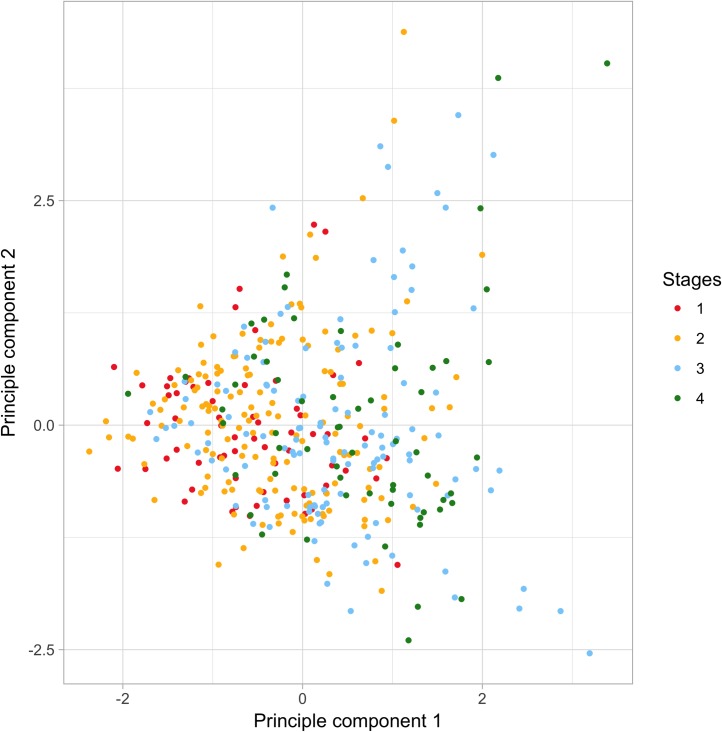
Principal component 1 and principle component 2 by cancer stage.

Principal component analysis result indicated that the first principal component (PC1) explained 16% of the variability, while PC2 explained 9.7% and PC3 explained 4.8% of the variability in the gene expression data in all the CRC samples. **Fig 2** illustrated PC1 vs. PC2 for all the CRC samples across four stages. The samples from these four stages do not appear to have distinct gene expression patterns.

### Gene annotation and network-based analysis

Network analysis showed that the top eleven genes map to multiple pathways such as meiotic synapsis and packaging of telomere ends, etc. (**S1 Table**). Ten of these 11 genes were linked to Gene Ontology (GO) terms such as nucleosome, DNA packaging complex and protein-DNA interactions (**S2 Table**). The protein complex-based gene set analysis showed that four genes were involved in H2AX complex II with q value of 5.72*10–5 (**S3 Table**). The enriched neighborhood based sets analysis of these 11 genes (**S1 Fig**) identified CDC like kinase 2 be connected with most genes (386 genes) in the neighborhood. *RNF4* and *RNF8* genes, in the same family as one of the top genes (RNF34), were also well-connected with multiple genes in pathways. Finally, the induced network module analysis identified several genes with gene protein interaction: *HIST2H4A*, *HIST3H2BB*, *LRCH4* and *NUDT6* (**S2 Fig**).

## Discussion

Using publically available data from TCGA, this study explored the gene expression differences across four stages of CRC. We found that eleven genes showed suggestive level of evidence for differential expression in a linear fashion. These genes map to multiple pathways and were linked to GO terms. Further, several few genes were enriched in protein complexes. However, a principal component analysis was not able to identify a molecular basis for the current CRC staging process. This might be due to the following: 1) due to the limitation of publically available data, our study was not able to compare the gene expression data from different CRC stages with a normal control; 2) the CRC staging system currently uses the size of lesion for staging, not molecular basis; and 3) the principal component analysis was able to cover only ~30% of the variance in the gene expression data. Such analysis has not been done previously in the literature.

Among the genes with suggestive level of significance, only a few had possible link with cancer in the literature. The gene with the strongest p value for differential expression by stage is ***NEK4*** gene, which encodes NIMA related kinase 4, a serine/threonine protein kinase required for normal entry info replicative senescence. In cell culture, suppression of NEK4 doubled the number of replications needed to reach senescence, reduced cellular reactions to double-stranded DNA damage in both recruitment of repair proteins and arresting of further cell divisions, and also reduced activity of the p53 tumor suppressor protein [18]. Our study suggested that the CRC patients in the higher stages have lower NEK4 gene expression compared to lower stages, this is consistent with the direction shown in tissue culture [18] that lower expression was associated with worse diagnosis.

***RNF34*** gene, which encodes ring finger protein 34, was first known and characterized as **hRFI** (human ring finger homologous to inhibitor of apoptosis protein type) in 2005, was shown to have anti-apoptotic properties [19], and later was shown to also play a role in regulation of p53 via ubiquitination and subsequent proteasomal degradation [20]. Overexpression of this gene was shown to confer the resistance to 5-fluorouracil-induced apoptosis in colorectal cancer cells via activation of NF-kappaB and upregulation of BCL-2 and BCL-XL [21]. In our study, ***RNF34*** had lower expression in those in the more advanced clinical stages of CRC patients. This seems indicate that more advanced CRC patients may be more sensitive to 5-fluorouacil treatment compared to patients in earlier stages, but this is outside the scope of our study. However, it is worth noting that 5-fluorouacil is currently recommended as one of the adjuvant chemotherapy agents for stage III and high-risk stage II colon cancer patients [22].

***HIST3H2BB*** and ***HIST2H4A***, both encoding histone proteins, were also among the top differentially expressed genes, increasing in expression with increasing cancer stages. Eukaryotic DNA that is not currently being replicated is stored in a wrapped and coiled form around four pairs of histone proteins that provide support for the coiled DNA. Histones are also sensitive to post-translational modification, such as acetylation and deacetylation, which the cells use to help regulate transcription [23]. A direct link to the role of increased histone protein expression isn’t clear, perhaps further examination of co-expression levels of histone acetyltransferases and deacetylases would suggest a link.

Members of the ***NUDT6*** gene family exhibit behaviors that include controlling the level of cellular metabolites and signaling compounds as well as degrading “potentially mutagenic” oxidized nucleotides” [24]. The trend of downregulation of this gene across cancers stages would indeed contribute to the ability of cancer cells to continue to grow, divide, and evade normal cellular precautions.

***LRCH4*** gene encodes leucine rich repeats and calponin homology domain containing 4, which is a protein that contains leucine-rich repeats at its amino terminus and that is known to be involved in ligand binding. ***AMIGO2***, which encodes adhesion molecule with Ig like domain 1, is a leucine-rich repeat family member. ***AMIGO2*** mRNA was found to be differentially expressed in near half of cancer vs. normal tissue from gastric adenocarcinoma patients [25]. In an antisense study, it was found that the inhibition of **AMIGO2** expression negatively impact tumor growth and altered chromosomal stability [25].

Our study has some limitations: 1). TCGA CRC data only included data on samples from cancer patients, therefore the only analysis we could perform was within cancer samples and using controls from a different source would bring too much confounding. 2) The data from TCGA had many field with missing information, such as medication information, which may be altering gene expression in some of the genes or loci of interest. Therefore, no meaningful analysis can be performed with the medication data.

In conclusion, our study identified several genes that might be associated with clinical stages of CRC. Our analysis also suggests that the current clinical staging might not have molecular basis according to the gene expression patterns.

## Supporting information

S1 Fig Result of the enriched neighborhood-based sets (NESTs). (PDF)

S2 Fig Induced network module analysis. (PDF)

S1 Table Enriched pathway-based sets. (PDF)

S2 Table Enriched gene ontology-based sets. (PDF)

S3 Table Enriched protein complex-based sets. (PDF)

## References

[1] Siegel RL, Miller KD, Jemal A. Cancer Statistics, 2017. CA Cancer J Clin. 2017;67(1):7–30. Epub 2017/01/05. doi: 10.3322/caac.21387 . 28055103 10.3322/caac.21387

[2] Amin MB, Greene FL, Edge SB, Compton CC, Gershenwald JE, Brookland RK, et al The Eighth Edition AJCC Cancer Staging Manual: Continuing to build a bridge from a population-based to a more "personalized" approach to cancer staging. CA Cancer J Clin. 2017;67(2):93–9. Epub 2017/01/17. doi: 10.3322/caac.21388 . 28094848 10.3322/caac.21388

[3] Hari DM, Leung AM, Lee JH, Sim MS, Vuong B, Chiu CG, et al AJCC Cancer Staging Manual 7th edition criteria for colon cancer: do the complex modifications improve prognostic assessment? J Am Coll Surg. 2013;217(2):181–90. Epub 2013/06/12. doi: 10.1016/j.jamcollsurg.2013.04.018 ; PubMed Central PMCID: PMCPMC4657944. 23768788 10.1016/j.jamcollsurg.2013.04.018 PMC4657944

[4] Fodde R. The APC gene in colorectal cancer. Eur J Cancer. 2002;38(7):867–71. . 11978510 10.1016/s0959-8049(02)00040-0

[5] Musulen E, Blanco I, Carrato C, Fernandez-Figueras MT, Pineda M, Capella G, et al Usefulness of epithelial cell adhesion molecule expression in the algorithmic approach to Lynch syndrome identification. Hum Pathol. 2013;44(3):412–6. Epub 2012/09/29. doi: 10.1016/j.humpath.2012.06.006 . 23026194 10.1016/j.humpath.2012.06.006

[6] Tutlewska K, Lubinski J, Kurzawski G. Germline deletions in the EPCAM gene as a cause of Lynch syndrome—literature review. Hered Cancer Clin Pract. 2013;11(1):9 Epub 2013/08/12. doi: 10.1186/1897-4287-11-9 ; PubMed Central PMCID: PMCPMC3765447. 23938213 10.1186/1897-4287-11-9 PMC3765447

[7] Reimers MS, Kuppen PJ, Lee M, Lopatin M, Tezcan H, Putter H, et al Validation of the 12-gene colon cancer recurrence score as a predictor of recurrence risk in stage II and III rectal cancer patients. J Natl Cancer Inst. 2014;106(11). Epub 2014/09/26. doi: 10.1093/jnci/dju269 . 25261968 10.1093/jnci/dju269

[8] Mármol I, Sánchez-de-Diego C, Pradilla Dieste A, Cerrada E, Rodriguez Yoldi MJ. Colorectal Carcinoma: A General Overview and Future Perspectives in Colorectal Cancer. Int J Mol Sci. 2017;18(1). Epub 2017/01/19. doi: 10.3390/ijms18010197 ; PubMed Central PMCID: PMCPMC5297828. 28106826 10.3390/ijms18010197 PMC5297828

[9] O'Connell MJ, Lavery I, Yothers G, Paik S, Clark-Langone KM, Lopatin M, et al Relationship between tumor gene expression and recurrence in four independent studies of patients with stage II/III colon cancer treated with surgery alone or surgery plus adjuvant fluorouracil plus leucovorin. J Clin Oncol. 2010;28(25):3937–44. Epub 2010/08/02. doi: 10.1200/JCO.2010.28.9538 ; PubMed Central PMCID: PMCPMC2940392. 20679606 10.1200/JCO.2010.28.9538 PMC2940392

[10] Lorenc Z, Waniczek D, Lorenc-Podgórska K, Krawczyk W, Domagała M, Majewski M, et al Profile of Expression of Genes Encoding Matrix Metallopeptidase 9 (MMP9), Matrix Metallopeptidase 28 (MMP28) and TIMP Metallopeptidase Inhibitor 1 (TIMP1) in Colorectal Cancer: Assessment of the Role in Diagnosis and Prognostication. Med Sci Monit. 2017;23:1305–11. Epub 2017/03/15. doi: 10.12659/MSM.901593 ; PubMed Central PMCID: PMCPMC5363457. 28293015 10.12659/MSM.901593 PMC5363457

[11] Network CGAR. Comprehensive genomic characterization defines human glioblastoma genes and core pathways. Nature. 2008;455(7216):1061–8. Epub 2008/09/04. doi: 10.1038/nature07385 ; PubMed Central PMCID: PMCPMC2671642. 18772890 10.1038/nature07385 PMC2671642

[12] Tomczak K, Czerwińska P, Wiznerowicz M. The Cancer Genome Atlas (TCGA): an immeasurable source of knowledge. Contemp Oncol (Pozn). 2015;19(1A):A68–77. doi: 10.5114/wo.2014.47136 ; PubMed Central PMCID: PMCPMC4322527. 25691825 10.5114/wo.2014.47136 PMC4322527

[13] Mortazavi A, Williams BA, McCue K, Schaeffer L, Wold B. Mapping and quantifying mammalian transcriptomes by RNA-Seq. Nat Methods. 2008;5(7):621–8. Epub 2008/05/30. doi: 10.1038/nmeth.1226 . 18516045 10.1038/nmeth.1226 PMC13303166

[14] Li B, Dewey CN. RSEM: accurate transcript quantification from RNA-Seq data with or without a reference genome. BMC Bioinformatics. 2011;12:323 Epub 2011/08/04. doi: 10.1186/1471-2105-12-323 ; PubMed Central PMCID: PMCPMC3163565. 21816040 10.1186/1471-2105-12-323 PMC3163565

[15] Huang DW, Sherman BT, Tan Q, Kir J, Liu D, Bryant D, et al DAVID Bioinformatics Resources: expanded annotation database and novel algorithms to better extract biology from large gene lists. Nucleic Acids Res. 2007;35(Web Server issue):W169–75. Epub 2007/06/18. doi: 10.1093/nar/gkm415 ; PubMed Central PMCID: PMCPMC1933169. 17576678 10.1093/nar/gkm415 PMC1933169

[16] Herwig R, Hardt C, Lienhard M, Kamburov A. Analyzing and interpreting genome data at the network level with ConsensusPathDB. Nat Protoc. 2016;11(10):1889–907. Epub 2016/09/08. doi: 10.1038/nprot.2016.117 . 27606777 10.1038/nprot.2016.117

[17] Kamburov A, Wierling C, Lehrach H, Herwig R. ConsensusPathDB—a database for integrating human functional interaction networks. Nucleic Acids Res. 2009;37(Database issue):D623–8. Epub 2008/10/21. doi: 10.1093/nar/gkn698 ; PubMed Central PMCID: PMCPMC2686562. 18940869 10.1093/nar/gkn698 PMC2686562

[18] Nguyen CL, Possemato R, Bauerlein EL, Xie A, Scully R, Hahn WC. Nek4 regulates entry into replicative senescence and the response to DNA damage in human fibroblasts. Mol Cell Biol. 2012;32(19):3963–77. Epub 2012/07/30. doi: 10.1128/MCB.00436-12 ; PubMed Central PMCID: PMCPMC3457524. 22851694 10.1128/MCB.00436-12 PMC3457524

[19] Konishi T, Sasaki S, Watanabe T, Kitayama J, Nagawa H. Overexpression of hRFI (human ring finger homologous to inhibitor of apoptosis protein type) inhibits death receptor-mediated apoptosis in colorectal cancer cells. Mol Cancer Ther. 2005;4(5):743–50. doi: 10.1158/1535-7163.MCT-05-0020 . 15897238 10.1158/1535-7163.MCT-05-0020

[20] Vousden KH, Prives C. Blinded by the Light: The Growing Complexity of p53. Cell. 2009;137(3):413–31. doi: 10.1016/j.cell.2009.04.037 . 19410540 10.1016/j.cell.2009.04.037

[21] Konishi T, Sasaki S, Watanabe T, Kitayama J, Nagawa H. Overexpression of hRFI inhibits 5-fluorouracil-induced apoptosis in colorectal cancer cells via activation of NF-kappaB and upregulation of BCL-2 and BCL-XL. Oncogene. 2006;25(22):3160–9. doi: 10.1038/sj.onc.1209342 . 16407826 10.1038/sj.onc.1209342

[22] Vogel JD, Eskicioglu C, Weiser MR, Feingold DL, Steele SR. The American Society of Colon and Rectal Surgeons Clinical Practice Guidelines for the Treatment of Colon Cancer. Dis Colon Rectum. 2017;60(10):999–1017. doi: 10.1097/DCR.0000000000000926 . 28891842 10.1097/DCR.0000000000000926

[23] Grunstein M. Histone acetylation in chromatin structure and transcription. Nature. 1997;389(6649):349–52. doi: 10.1038/38664 . 9311776 10.1038/38664

[24] McLennan AG. The Nudix hydrolase superfamily. Cell Mol Life Sci. 2006;63(2):123–43. doi: 10.1007/s00018-005-5386-7 . 16378245 10.1007/s00018-005-5386-7 PMC11136074

[25] Rabenau KE, O'Toole JM, Bassi R, Kotanides H, Witte L, Ludwig DL, et al DEGA/AMIGO-2, a leucine-rich repeat family member, differentially expressed in human gastric adenocarcinoma: effects on ploidy, chromosomal stability, cell adhesion/migration and tumorigenicity. Oncogene. 2004;23(29):5056–67. doi: 10.1038/sj.onc.1207681 . 15107827 10.1038/sj.onc.1207681

