# Supplementary figures and images for: Colorectal cancer stages transcriptome analysis
Source: PLoS One. 2017 Nov 28;12(11):e0188697. doi: 10.1371/journal.pone.0188697 (PMC5705125; doi:10.1371/journal.pone.0188697)

### S1 Fig. Result of the enriched neighborhood-based sets (NESTs)

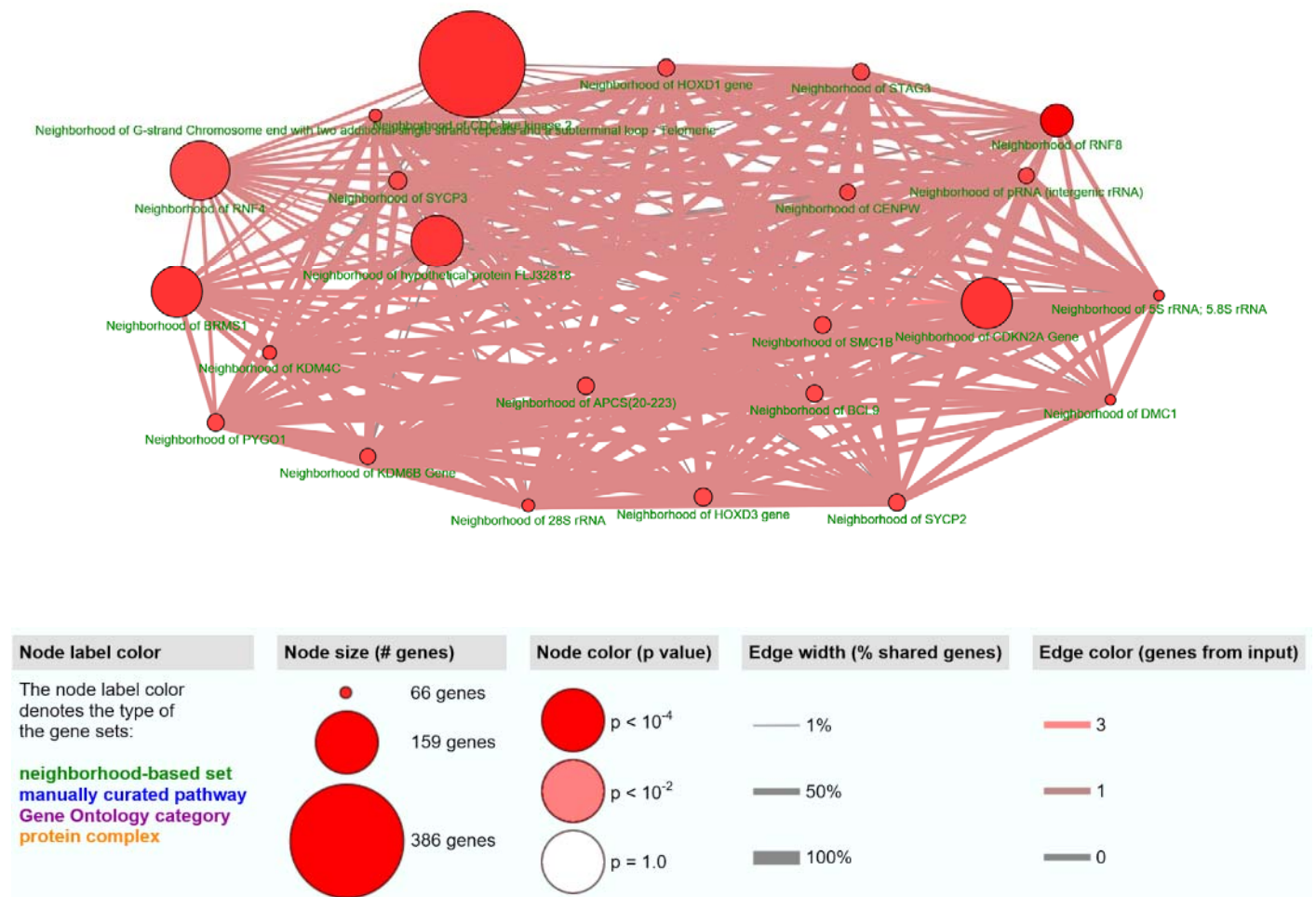

Supplement: S1 Fig — (PDF) [file pone.0188697.s001.pdf]

S2 Fig. Induced Network Module Analysis

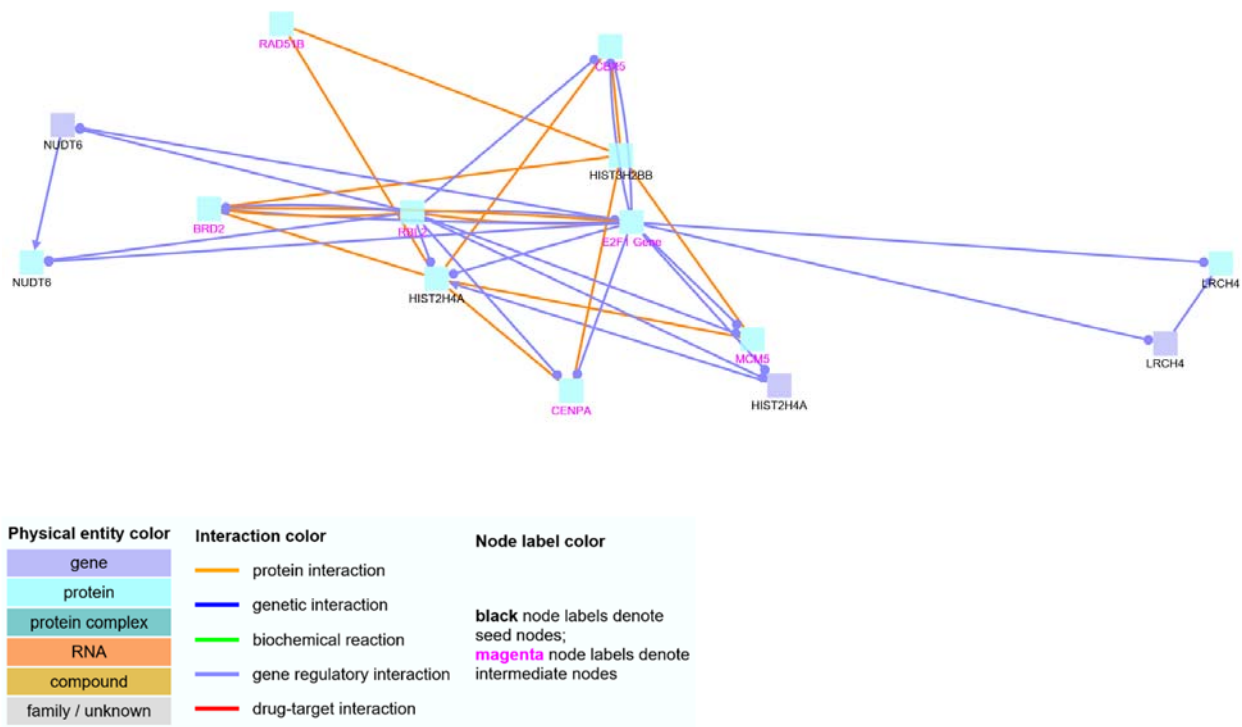

Supplement: S2 Fig — (PDF) [file pone.0188697.s002.pdf]
