# Supplementary material for: Colorectal cancer stages transcriptome analysis
Source: PLoS One. 2017 Nov 28;12(11):e0188697. doi: 10.1371/journal.pone.0188697 (PMC5705125; doi:10.1371/journal.pone.0188697)
Supplement: S1 Table — (PDF) [file pone.0188697.s003.pdf]

## S1 Table.

5 genes (50.0%) from the input list are present in at least one pathway.
The total number of genes present in at least one pathway and identifiable by 'hgnc-symbol' IDs is **12655**.

| select all none | pathway name | set size | candidates contained | p-value | q-value | pathway source |
|---|---|---|---|---|---|---|
| ☐ | Meiotic synapsis | 48 | 2 (4.3%) | 0.000134 | 0.00153 | Reactome |
| ☐ | Packaging Of Telomere Ends | 54 | 2 (3.8%) | 0.000171 | 0.00153 | Reactome |
| ☐ | Meiotic recombination | 65 | 2 (3.1%) | 0.000249 | 0.00153 | Reactome |
| ☐ | RNA Polymerase I Promoter Opening | 66 | 2 (3.1%) | 0.000257 | 0.00153 | Reactome |
| ☐ | DNA methylation | 68 | 2 (3.0%) | 0.000273 | 0.00153 | Reactome |
| ☐ | Activated PKN1 stimulates transcription of AR (androgen receptor) regulated genes KLK2 and KLK3 | 70 | 2 (2.9%) | 0.00029 | 0.00153 | Reactome |
| ☐ | Nonhomologous End-Joining (NHEJ) | 71 | 2 (2.9%) | 0.000298 | 0.00153 | Reactome |
| ☐ | SIRT1 negatively regulates rRNA Expression | 72 | 2 (2.8%) | 0.000307 | 0.00153 | Reactome |
| ☐ | Condensation of Prophase Chromosomes | 77 | 2 (2.6%) | 0.000352 | 0.00153 | Reactome |
| ☐ | PRC2 methylates histones and DNA | 77 | 2 (2.6%) | 0.000352 | 0.00153 | Reactome |
| ☐ | Recruitment and ATM-mediated phosphorylation of repair and signaling proteins at DNA double strand breaks | 77 | 2 (2.6%) | 0.000352 | 0.00153 | Reactome |
| ☐ | DNA Double Strand Break Response | 78 | 2 (2.6%) | 0.000361 | 0.00153 | Reactome |
| ☐ | ERCC6 (CSB) and EHMT2 (G9a) positively regulate rRNA expression | 79 | 2 (2.6%) | 0.000371 | 0.00153 | Reactome |
| ☐ | Meiosis | 79 | 2 (2.6%) | 0.000371 | 0.00153 | Reactome |
| ☐ | Amyloid fiber formation | 83 | 2 (2.4%) | 0.00041 | 0.00153 | Reactome |
| ☐ | Telomere Maintenance | 85 | 2 (2.4%) | 0.00043 | 0.00153 | Reactome |
| ☐ | RNA Polymerase I Chain Elongation | 94 | 2 (2.2%) | 0.000527 | 0.00153 | Reactome |
| ☐ | HDACs deacetylate histones | 94 | 2 (2.1%) | 0.000538 | 0.00153 | Reactome |
