# Supplementary material for: Colorectal cancer stages transcriptome analysis
Source: PLoS One. 2017 Nov 28;12(11):e0188697. doi: 10.1371/journal.pone.0188697 (PMC5705125; doi:10.1371/journal.pone.0188697)
Supplement: S2 Table — (PDF) [file pone.0188697.s004.pdf]

S2 Table.

10 genes (100.0%) from the input list are present in at least one GO category.
The total number of genes present in at least one GO category and identifiable by 'hgnc-symbol' IDs is **18839**.

select all none	gene ontology term	category, level	set size	candidates contained	p-value	q-value
☐	GO:0000786 nucleosome	CC 3	**106**	**2 (1.9%)**	0.00134	0.0121
☐	GO:0044815 DNA packaging complex	CC 2	**112**	**2 (1.8%)**	0.0015	0.021
☐	GO:0034728 nucleosome organization	BP 5	**173**	**2 (1.2%)**	0.00355	0.0373
☐	GO:0032993 protein-DNA complex	CC 2	**180**	**2 (1.1%)**	0.00384	0.0269
☐	GO:0006333 chromatin assembly or disassembly	BP 5	**188**	**2 (1.1%)**	0.00419	0.0373
☐	GO:0065004 protein-DNA complex assembly	BP 5	**224**	**2 (0.9%)**	0.0059	0.0373
☐	GO:0071824 protein-DNA complex subunit organization	BP 4	**251**	**2 (0.8%)**	0.00736	0.243
☐	GO:0071103 DNA conformation change	BP 5	**287**	**2 (0.7%)**	0.00954	0.0453
