# Supplementary material for: Colorectal cancer stages transcriptome analysis
Source: PLoS One. 2017 Nov 28;12(11):e0188697. doi: 10.1371/journal.pone.0188697 (PMC5705125; doi:10.1371/journal.pone.0188697)
Supplement: S3 Table — (PDF) [file pone.0188697.s005.pdf]

S3 Table.

Enriched protein complex-based sets (download)

4 genes (40.0%) from the input list are present in at least one protein complex.
The total number of genes present in at least one protein complex and identifiable by 'hgnc-symbol' IDs is **9211**.

select all none	complex name	set size	candidates contained	p-value	q-value	complex source
☐	H2AX complex II	23	2 (8.7%)	3.57e-05	5.72e-05	CORUM
☐	H2AX complex, isolated from cells without IR exposure	29	2 (6.9%)	5.72e-05	5.72e-05	CORUM
